# The geometrical precision of virtual bone models derived from clinical computed tomography data for forensic anthropology

**DOI:** 10.1007/s00414-017-1548-z

**Published:** 2017-02-10

**Authors:** Kerri L. Colman, Johannes G. G. Dobbe, Kyra E. Stull, Jan M. Ruijter, Roelof-Jan Oostra, Rick R. van Rijn, Alie E. van der Merwe, Hans H. de Boer, Geert J. Streekstra

**Affiliations:** 1 Department of Medical Biology, Academic Medical Centre, University of Amsterdam, P.O. Box 22660, 1100 DD Amsterdam, The Netherlands; 2 Department of Biomedical Engineering and Physics, Academic Medical Centre, University of Amsterdam, P.O. Box 22660, 1100 DD Amsterdam, The Netherlands; 3 Department of Anthropology, University of Nevada, Reno, 1664 N. Virginia Street, Reno, NV 89557 USA; 4 Department of Anatomy, University of Pretoria, Private Bag x323, Arcadia, 0081 Pretoria South Africa; 5 Department of Radiology, Academic Medical Centre, University of Amsterdam, P.O. Box 22660, 1100 DD Amsterdam, The Netherlands; 6 Department of Pathology, Academic Medical Centre, University of Amsterdam, P.O. Box 22660, 1100 DD Amsterdam, The Netherlands

**Keywords:** Radiology, Segmentation, Precision, Methodology, Kolmogorov–Smirnov, Pelvis

## Abstract

Almost all European countries lack contemporary skeletal collections for the development and validation of forensic anthropological methods. Furthermore, legal, ethical and practical considerations hinder the development of skeletal collections. A virtual skeletal database derived from clinical computed tomography (CT) scans provides a potential solution. However, clinical CT scans are typically generated with varying settings. This study investigates the effects of image segmentation and varying imaging conditions on the precision of virtual modelled pelves. An adult human cadaver was scanned using varying imaging conditions, such as scanner type and standard patient scanning protocol, slice thickness and exposure level. The pelvis was segmented from the various CT images resulting in virtually modelled pelves. The precision of the virtual modelling was determined per polygon mesh point. The fraction of mesh points resulting in point-to-point distance variations of 2 mm or less (95% confidence interval (CI)) was reported. Colour mapping was used to visualise modelling variability. At almost all (>97%) locations across the pelvis, the point-to-point distance variation is less than 2 mm (CI = 95%). In >91% of the locations, the point-to-point distance variation was less than 1 mm (CI = 95%). This indicates that the geometric variability of the virtual pelvis as a result of segmentation and imaging conditions rarely exceeds the generally accepted linear error of 2 mm. Colour mapping shows that areas with large variability are predominantly joint surfaces. Therefore, results indicate that segmented bone elements from patient-derived CT scans are a sufficiently precise source for creating a virtual skeletal database.

**Electronic supplementary material:**

The online version of this article (doi:10.1007/s00414-017-1548-z) contains supplementary material, which is available to authorized users.

## Introduction

The development and validation of forensic anthropological methods rely on the availability of contemporary, population-specific skeletal collections of considerable size [1–5]. Examples hereof are skeletal collections such as the Bass, Terry, Hamman Todd, Pretoria Bone and Raymond Dart collection [6–10]. Each of these collections suffers from its unique blend of selection bias. For instance, the Bass collection holds predominantly white males between the ages of 35 and 85 years [11], while the Pretoria Bone Collection holds a majority of black males between the ages of 30 and 80 years [6]. Additionally, in most skeletal collections, females are underrepresented and the collection’s demographics are not reflective of the larger population [12, 13]. Despite these biases, these skeletal collections serve as modern collections from which biological profile methods are developed.

The vast majority of European countries lack modern skeletal collections [14–23]. Many European institutions have a rich collection of archaeological specimens, but secular changes disqualify archaeological material and cemetery populations, in general, as reference populations for forensic purposes [24]. Furthermore, the development of modern representative skeletal collections is hindered by legal, ethical and practical considerations [25].

A possible solution to this problem is provided by the vast amount of data that is routinely generated in the hospital setting by medical imaging techniques, such as computed tomography (CT) scans. Such patient-derived CT scans could form the basis of a virtual skeletal database. Patient-derived, anonymized CT images are available in abundance, and a large, representative skeletal collection could thus be developed in a relatively short period of time. In addition, a virtual skeletal database does not require time-consuming and labour-intensive skeletal processing techniques (e.g. maceration) or physical storage space.

The success of a virtual skeletal database depends on the precise modelling of virtual skeletal elements from the CT images and the accuracy with which virtually modelled skeletal elements represent their dry bone counterparts. Previous studies have investigated the accuracy of virtual bones, using CT images of cadavers or dry bone elements, with promising results [26–29]. However, the CT scans in these studies are typically conducted under ideal conditions. For example, protocols were not limited by exposure level (mAs) restrictions and, in the case of dry bone elements, without surrounding soft tissue. Both exposure level (mAs) and the biological composition of the scanned subject influence the quality and noise level of the CT image and therefore theoretically could affect the modelled virtual bone [30]. Conversely, patient-derived CT scans are conducted under varying imaging conditions, such as variations in the acquisition parameters (i.e. exposure level, slice thickness, increment level, reconstruction filter) and in the type/brand of scanner used. These variations may influence the geometric variability of the virtual skeletal elements. Also intra- and inter-observer variation in the segmentation process, which provides the virtual models, may add geometric variability to the virtual model.

Knowledge on the extent to which these sources of variability affect the geometric variability (i.e. precision) of the virtual model serves as a basis for the inclusion and exclusion criteria of CT scans for setting up a virtual skeletal database. Also, this knowledge may facilitate an increase in research using 3D models from patient-derived CT scans for the development of forensic anthropological methods [31–35].

Therefore, this study aims to investigate the effects of image segmentation (intra- and inter-observer variability) and varying imaging conditions, such as scanner type with the associated standard patient scanning protocol, slice thickness and exposure level on the geometric variability of 3D virtual models of the human pelvis. The pelvis was specifically selected because of its multifaceted morphology, its relevance in forensic anthropological sex estimation techniques and for its low signal-to-noise ratio in CT scans, which adds complexity to the virtual modelling process.

## Materials and methods

The current study consists of several imaging experiments, all using the same embalmed human cadaver (63-year-old male) from the body donation program in the Department of Medical Biology of the Academic Medical Centre (AMC), University of Amsterdam, the Netherlands. The cadaver was scanned multiple times on two comparable CT scanners, a Philips Brilliance 64 (Philips Medical Systems, Best, the Netherlands) and a Siemens Sensation 64 (Siemens Healthineers, Erlangen, Germany), using variations of standard patient imaging protocols. To include variability in the scanning process, including the random effect of Poisson noise, quintuplicate CT scans of the same pelvis were made, without repositioning the pelvis between subsequent scans. In each experiment, only one source of variability was altered at a time, enabling the interpretation of each parameter’s influence compared to the other varying imaging conditions. While the imaging settings of the CT scanner remain constant except for the one variable being evaluated, there are some imaging parameters, such as scanner type, that are always incorporated into the overall variability. Image segmentation of the five CT images yielded five virtually modelled pelves of the same physical pelvis, for each source of variability. A total of five sources of variability were studied, namely intra-observer variability, inter-observer variability, scanner type, slice thickness and exposure level.

Each source of variability was evaluated by comparing two quintuplicate sets of virtual bone models, derived from the CT images.

### Creating a virtual pelvis model from CT images

The pelvic bone was segmented out of each CT image using in-house segmentation software [36]. Image segmentation is a semi-automatic procedure that results in a polygon mesh, consisting of thousands of points representing the virtual bone model. Segmentation starts with a threshold-connected region-growing algorithm [37] in which the user can interactively adapt the threshold until an optimal number of bone voxels are selected that gives no or minimal leakage to neighbouring structures. Structures that are missed in this procedure can then be added manually using an on-screen brush. A binary closing algorithm [38] helps to fill residual holes and to close the outline. Next, a Laplacian level-set growth algorithm [37] is used to advance voxels towards the edges of the bone. A distance map is finally used to extract a polygon mesh at the zero-level using a marching cubes algorithm [39].

### Sources of variability

The following two scanners with their standard clinical scanning protocols, and variations thereof, were used to study the effects of the various sources of variability:

1. Scanner type: Philips Brilliance 64 (Philips Medical Systems, Best, The Netherlands).
  Standard scanning protocol: 120 kV, 150 mAs, slice thickness 0.9 mm, increment 0.45 mm, reconstruction kernel D.
2. Scanner type: Siemens Sensation 64 (Siemens Healthineers, Erlangen, Germany).
  Standard scanning protocol: 120 kV, 200 mAs, slice thickness 1 mm, increment 1 mm, reconstruction kernel B60f.

The sources of variability, associated experiments and the scanning protocols are listed in Table 1.

**Table 1:** Sources of variability, the associated experiments, and the scanning protocols used

Source of variability	Experiments
*Image Segmentation*	
Intra-observer variation:	Round 1 vs Round 2^a^
Inter-observer variation:	Observer 1 vs. Observer 2^a^
*Imaging Conditions*	
Scanner type	Philips^a^ vs. Siemens^b^
Slice thickness	0.9 mm^a^ vs. 3.0 mm^c^
Exposure level (mAs)	100%^a^ vs. 50%^c^
	100%^b^ vs. 50%^d^

^a^Philips Brilliance 64 standard patient protocol (120 kV, 150 mAs, slice thickness 0.9 mm, increment 0.45 mm, reconstruction kernel D)

^b^Siemens Sensation 64 standard patient protocol (120 kV, 200 mAs, slice thickness 1 mm, increment 1 mm, reconstruction kernel B60f)

^c^Changes with respect to the Philips Brilliance 64 standard patient scanning protocol

^d^Changes with respect to the Siemens Sensation 64 standard patient scanning protocol

#### Geometric variability due to image segmentation

##### Intra-observer variability

One observer segmented the same set of the quintuplicate CT images twice (Round 1 and Round 2). The CT images were acquired by the Philips Brilliance 64 using its standard patient protocol. A 2-month gap between each segmentation session was used to try eliminate recognition.

##### Inter-observer variability

Two observers each segmented the same quintuplicate CT images acquired by the Philips Brilliance 64 using its standard patient protocol.

#### Geometric variability due to varying imaging conditions

##### Scanner type with their associated standard patient scanning protocol

One observer segmented two sets of quintuplicate CT images that were acquired by the Siemens Sensation 64 and the Philips Brilliance 64, using their standard patient imaging protocols. Note that the differences in the standard patient imaging protocols include exposure level (mAs), slice thickness, increment level and reconstruction filter.

##### Slice thickness

One observer segmented two sets of quintuplicate CT images that were acquired by the Philips Brilliance 64: one set with standard slice thickness (0.9 mm) (standard for bone scans) and one set with an increased slice thickness (3.0 mm) (standard for abdominal scans).

##### Exposure level (mAs)

For each scanner type, one observer segmented two sets of quintuplicate CT images acquired with full exposure levels (100%) and with halved (50%) exposure levels.

### Quantifying and visualizing geometric variability

The variability in the process of virtual modelling, due to image segmentation and imaging conditions, results in small differences between the points of each set of polygon meshes that make up the virtual model. To quantify the geometric variability between these polygon meshes in each quintuplicate set of models, the standard deviation (SD) for each single-point was calculated.

To calculate the single-point SD value, one of the five polygon meshes served as a reference, while the nearest-neighbour distance to a corresponding point in each of the other four polygon meshes was determined. These four distances were used to calculate the single-point SD value. Since the selection of the reference pelvis model may influence the single-point SD value, this procedure was repeated with every polygon mesh acting as a reference, which yielded five single-point SD values per mesh point location. The five single-point SD values per mesh point were averaged to obtain balanced single-point SD values that were independent of the polygon mesh used as a reference. These balanced single-point SD values were used for further statistical analysis and to visualize regions of high and low variation using colour mapping.

It should be noted that all segmentations result in different polygon meshes, with different numbers of points. This means that there are no real ‘corresponding’ points available to determine SD values. Selecting the nearest-neighbouring points in this study as an alternative to find a distance measure may reduce the variability and may therefore underestimate the SD value per point, especially if the polygon surface is influenced by noise. Since the level-set segmentation growth algorithm features image filtering, and therefore smoothing of each neighbouring polygon surface, this effect is considered to be too small to affect the outcomes of the study.

#### Statistical analysis

The distributions of SD values obtained from the virtual models, for each source of variability, were compared using a Kolmogorov–Smirnov test. A Kolmogorov–Smirnov test quantifies the difference of the SD distributions by determining the largest distance between the cumulative distributions of the two evaluated sources of variability. The test results in a *D*-value, which represents the single, largest geometric variability between the cumulative SD distributions of two sets of pelves, and at which SD level this occurs. The *D*-value ranges between 0 and 1, with 1 indicating a maximal difference and 0 indicating no difference. For the associated *p* value, a value of <0.05 is used to indicate a significant difference between the distributions.

#### Geometric variability of the virtual model

Traditional anthropology considers a threshold of 2 mm an acceptable measurement error for linear measurements [26]. Virtual models cannot exceed this level of error in order to be considered suitable replacements or substitutes for skeletal remains. In order to achieve this, the point-to-point distance variation of two polygon mesh points should have a 95% confidence interval (CI) of 2 mm or less. Specifically, the error propagation for difference in distances of two measurements indicates that each single-point should not have an SD value exceeding 0.7 mm.

While 2 mm is the upper limit for point-to-point distance variation for forensic anthropological purposes, other values are important for interpretation. Specifically, a balanced single-point SD value of 0.35, 0.175 and 0.07 mm results in a point-to-point distance variation with a 95% CI of 1, 0.5 and 0.25 mm, respectively.

The precision of the virtual model, per source of variability, was determined by categorizing the fractions of balanced single-point SD values of each polygon mesh. Virtual models with larger regions of high single-point SD values (>0.7 mm) are considered to be less precise.

#### Practical significance for forensic anthropological purposes

Statistical significance is required to establish differences. However, these differences may occur in locations of limited importance for forensic anthropological purposes. We illustrate the practical significance of our results for forensic anthropological purposes by colour maps. These visualize the modelling variation by colour mapping the balanced single-point SD values of each polygon mesh. When creating the colour map, the first reference mesh was selected for visualization and a balanced SD value was assigned to each mesh point. The computation of the SD values was calculated per source of variability; therefore, there are two colour maps, one per the two sources of variability being compared. The colour thresholds are 0.07, 0.175, 0.35, 0.7 and >0.7 mm.

## Results

### Statistical analysis

**Fig. 1:**
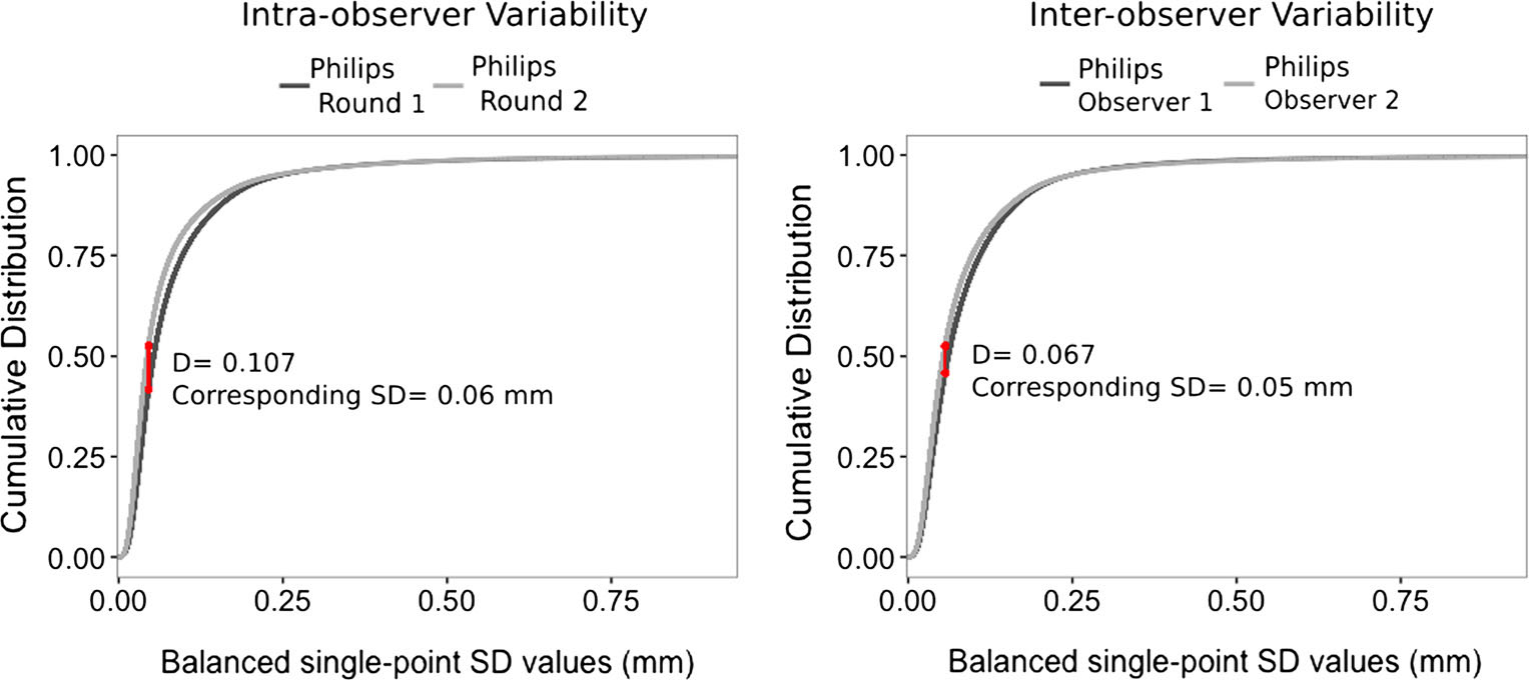
Difference in the cumulative distribution of single-point SD values, due to intra- and inter-observer variability. Visible in *red* is the location of the largest distance between the two cumulative distributions. The distances *D* = 0.107 and *D* = 0.067 correspond with a point-to-point distance variation of less than 0.25 mm (CI = 95%)

**Fig. 2:**
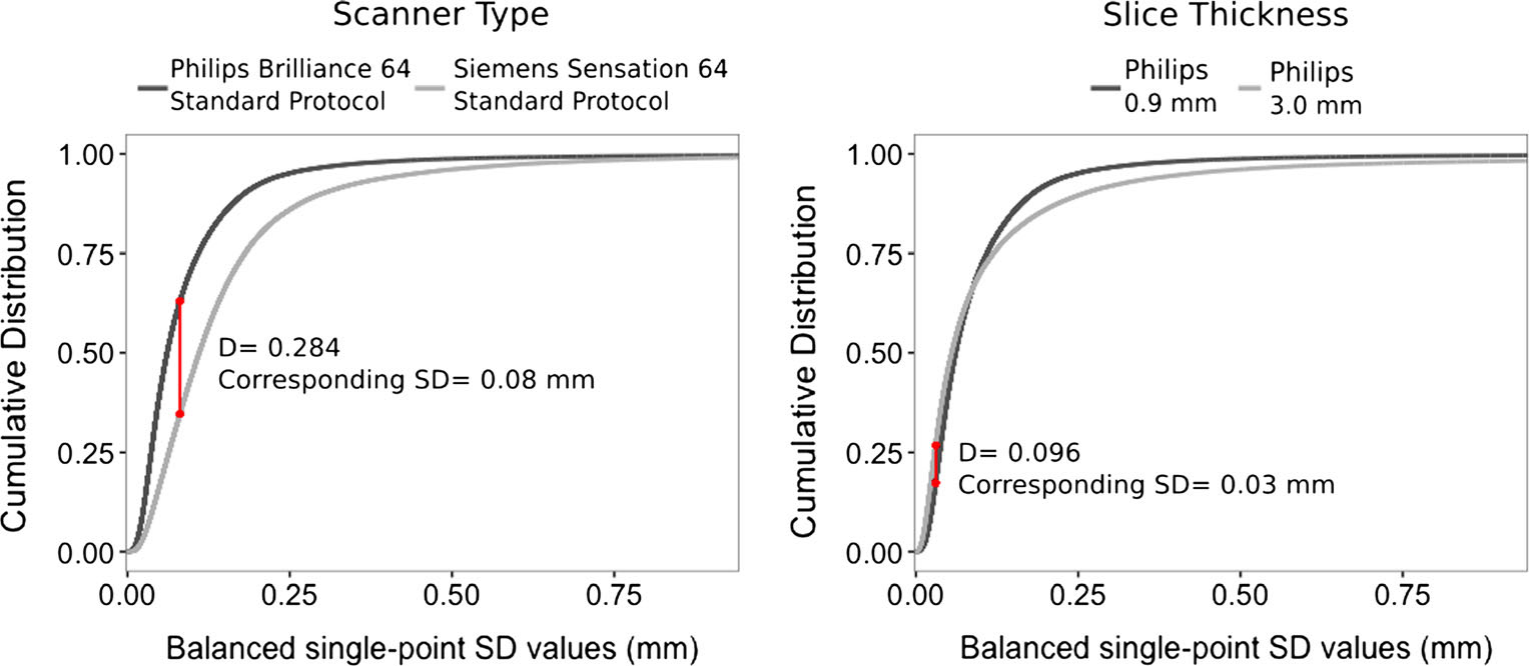
Difference in the cumulative distribution of single-point SD values, due to scanner type with their associated standard patient scanning protocol, and slice thickness variability. Visible in *red* is the location of the largest distance between the two cumulative distributions. The distances *D* = 0.2084 and *D* = 0.096 correspond with a point-to-point distance variation of less than 1 and 0.25 mm (CI = 95%), respectively

**Fig. 3:**
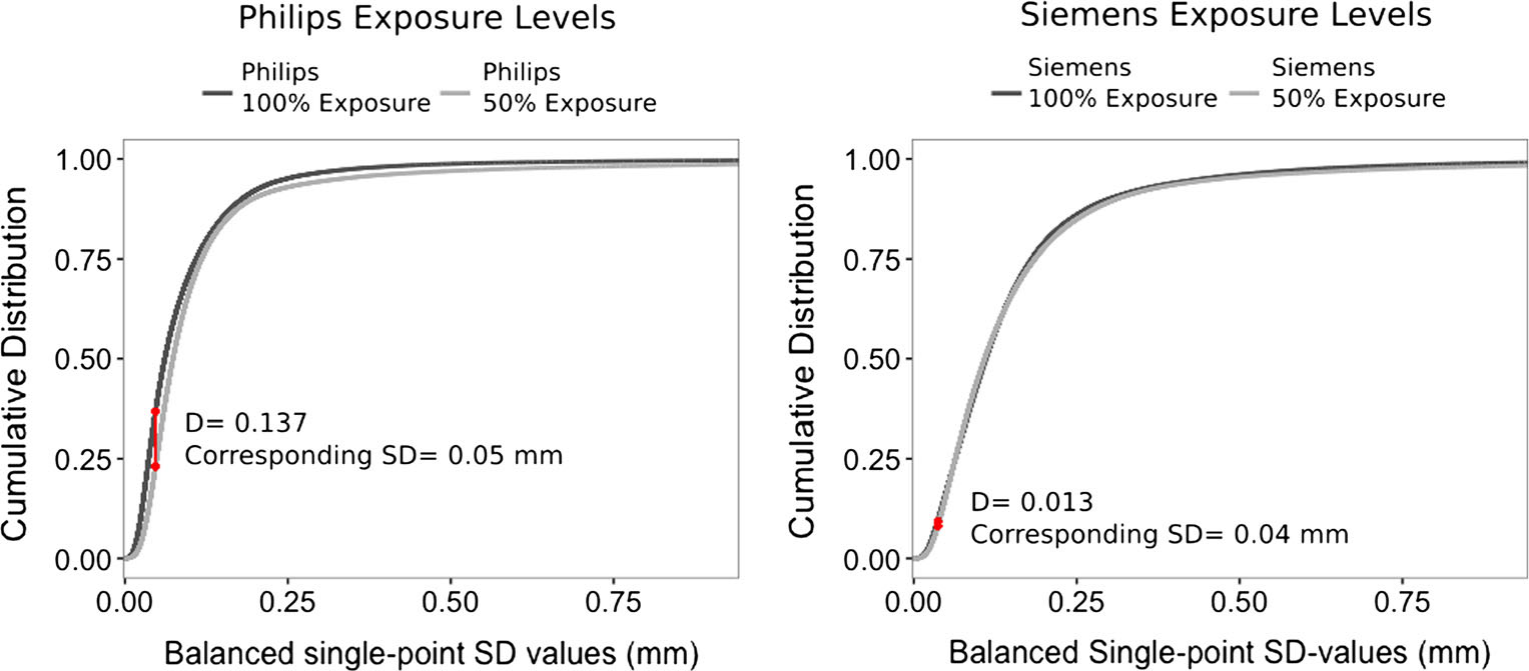
Difference in the cumulative distribution of single-point SD values, due to exposure level variability. Visible in *red* is the location of the largest distance between the two cumulative distributions. The distances *D* = 0.137 and *D* = 0.013 correspond with a point-to-point distance variation of less than 0.25 mm (CI = 95%)

The cumulative distributions of the single-point SD values obtained from the Kolmogorov–Smirnov test are visualized in Figs. 1, 2 and 3. From a statistical point of view, the cumulative distribution per source of variability differs significantly (all *p* values <0.001). However, the figures demonstrate that the largest differences (*D*-values) occur at single-point SD values that correspond to a point-to-point distance variation well below the 2 mm (CI = 95%) threshold. The largest *D*-value (0.284), associated with the scanner type with their standard patient scanning protocols, occurs at an SD value that corresponds to a point-to-point distance variation of less than 0.5 mm (CI = 95%). The smallest *D*-value (0.013) is associated with the difference in exposure levels (100 vs 50%) for the Siemens Sensation 64 and occurs at an SD value that corresponds to a point-to-point distance variation of less than 0.25 mm (CI = 95%).

### Geometric variability of the virtual model

For all sources of variability, more than 97% of the single-point SD values lie below the threshold of 0.7 mm and thus result in point-to-point distance variation of 2 mm (CI = 95%) or less (Figs. 1, 2 and 3, Table 2). Additionally, more than 91% of the single-point SD values lie below the 0.35 mm threshold, resulting in point-to-point distance variations of less than 1 mm (CI = 95%), which is well below the accepted error level of 2 mm in traditional forensic anthropology.

**Table 2:** Fractions of SD values (mm) that fall below each single-point SD threshold, per source of variability

Point-to-point distance (95% CI)	0.25 mm	0.5 mm	1 mm	2 mm	>2 mm
Single-point SD threshold	<0.07 mm	<0.175 mm	<0.35 mm	<0.7 mm	>0.7 mm
*Intra-observer variability*					
Philips observer 1^a^	.564	.893	.975	.993	.007
Philips observer 2	.626	.901	.973	.993	.007
*Inter-observer variability*					
Philips round 1^a^	.626	.901	.973	.993	.007
Philips round 2	.708	.916	.972	.994	.006
*Scanner type*					
Philips Brilliance 64^a^	.564	.893	.975	.993	.007
Siemens Sensation 64^b^	.285	.735	.924	.981	.019
*Slice thickness*					
Philips 0.9 mm^a^	.564	.893	.975	.993	.007
Philips 3 mm	.588	.835	.935	.975	.025
*Philips exposure levels*					
Philips exposure 100%^a^	.564	.893	.975	.993	.007
Philips exposure 50%	.468	.878	.953	.980	.020
*Siemens exposure levels*					
Siemens exposure 100%^b^	.285	.735	.924	.981	.019
Siemens exposure 50%	.290	.726	.919	.973	.027

^a^Repeated data used for multiple Philips comparisons

^b^Repeated data used for multiple Siemens comparisons

On average, half of the polygon mesh points have a point-to-point distance variation of 0.25 mm (CI = 95%) or less. This is with exception of CT images generated with the Siemens Sensation 64 scanner and its standard patient scanning protocol, where this only holds true for 30% of the mesh points. As expected, as the point-to-point distance variation decreases, the fraction of polygon mesh points within that increment also decreases.

### Practical significance for forensic anthropological purposes

**Fig. 4:**
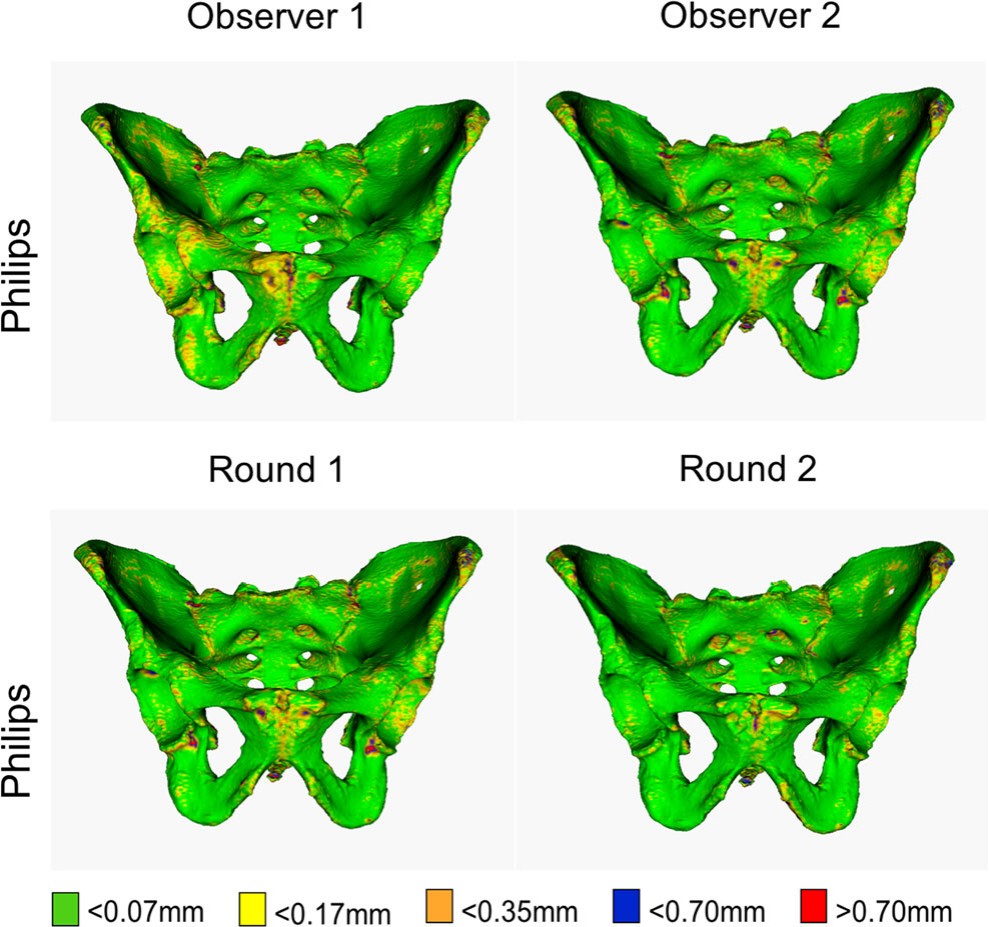
Colour maps showing geometric variability due to intra-observer variability and inter-observer variability. Single-point SD values of 0.07, 0.175, and 0.35 mm result with a point-to-point distance variations of 0.25, 0.5, and 1 mm (CI = 95%), respectively. The maps were obtained by segmenting quintuplicate CT scans of the pelvis from different scans and by quantifying the variability in point positions along the pelvic surface

**Fig. 5:**
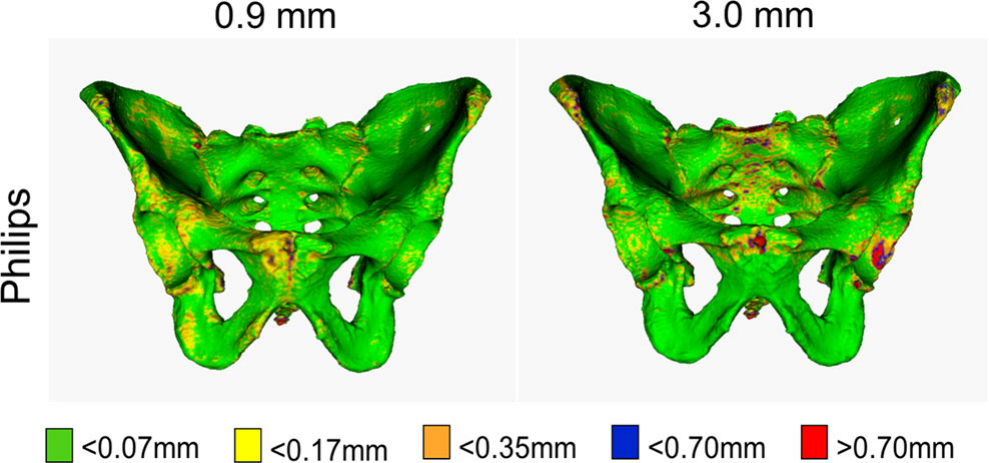
Colour maps showing geometric variability due to slice thickness. Single-point SD values of 0.07, 0.175, and 0.35 mm result with a point-to-point distance variations of 0.25, 0.5, and 1 mm (CI = 95%), respectively. These maps were obtained by segmenting quintuplicate CT scans of the pelvis from different scans (one observer) and by quantifying the variability in point positions along the pelvic surface

**Fig. 6:**
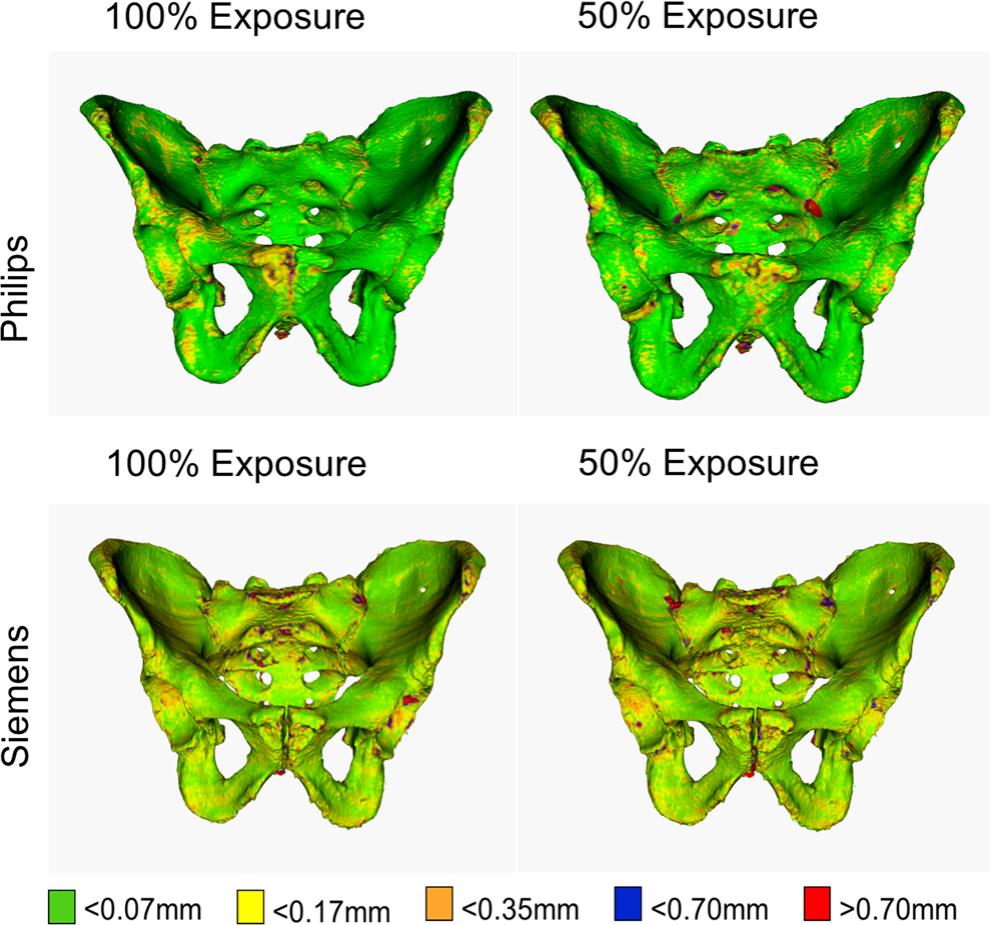
Colour maps showing geometric variability due to scanner type with their associated standard patient scanning protocol and exposure levels. Single-point SD values of 0.07, 0.175, and 0.35 mm result with a point-to-point distance variations of 0.25, 0.5, and 1 mm (CI = 95%), respectively. These maps were obtained by segmenting quintuplicate CT scans of the pelvis from different scans (one observer) and by quantifying the variability in point positions along the pelvic surface

Figures 4, 5 and 6 and supplemental material 1–12 display the distribution of the single-point SD values, per source of variability, on a reference pelvis. Each colour map displays a similar pattern. Joint surfaces (i.e. the sacro-iliac joint and the pubic symphysis), the posterior surface of the sacrum, the tip of the coccyx and areas typically associated with osteophytes have single-point SD values larger than 0.7 mm. Moreover, colour maps created from CT images acquired by the Siemens Sensation 64 scanner show a slightly larger region with single-point SD values larger than 0.7 mm. These areas are specifically in the acetabulum and the anterior surface of the sacrum, as visualized in Fig. 6.

## Discussion

Our results show that despite image segmentation and varying imaging conditions, all virtual models were sufficiently precise in most surface regions of the pelvis. At almost all (>97%) locations across the pelvis, the point-to-point distance variation is less than 2 mm (CI = 95%). Additionally, in more than 91% of the locations, the point-to-point distance variation is less than 1 mm (CI = 95%). This indicates that the geometric variability of the virtual pelvis as a result of segmentation and imaging conditions rarely exceeds the generally accepted point-to-point threshold of 2 mm [26]. Importantly, human error in segmentation is likely playing more of a role in imprecision than the varying imaging conditions.

Virtual models created from CT images with full exposure levels or minimal slice thickness produce virtual bone models that are more precise, as seen by the higher fraction of lower single-point SD values. Lower exposure levels result in higher noise levels and lower image quality, which influences the precision of the image segmentation process and ultimately yields larger variability in specific regions after virtual bone modelling. However, the difference between these virtual models and those produced with halved exposure levels or increased slice thickness are below the above-mentioned 2 mm threshold and are therefore considered negligible.

The variability incorporated in this study is believed to be representative of a worst-case scenario, both for biological and radiographic aspects. For example, pelvic scans are hampered by image noise due to a relatively large amount of attenuation by surrounding soft tissue. Additionally, the cadaver used in this study was of an older individual and subsequently probably had a lower bone density and more osteophytes than a young- or middle-aged individual. Considering all of the above, the authors agree that images generated of other skeletal elements as part of standard clinical CT scanning will likely yield virtual bone models with a higher precision than that presented in this study.

The use of only one pelvis in this study could be perceived as a limitation; however, by only incorporating one pelvis into the research design, additional sources of error, which may result from variation in body composition, were excluded. This allowed for unobscured analysis of the geometrical precision of the virtual bone model, which was the aim of this study.

Surface modelling rather than volume rendering techniques were applied in this study. This technique allows for the conversion of volume data into polygon mesh points that accurately represent the anatomical surface of an object [40]. By quantifying the error in the polygon mesh points, a foundation was laid for researchers to incorporate different and innovative techniques/methodologies to continually enhance the field of forensic anthropology. For example, the lack of measurement error in the polygon mesh points permits the possibility to conduct shape-fitting analyses and automate measurements on patient data. Shape fitting is considered less sensitive to small modelling variations (intra-/inter-observer, noise), which ultimately increases the distinctive power of detecting morphological features [41].

## Application to forensic anthropology

The findings from this research add to the current shift in forensic anthropology towards using virtual skeletal databases and virtual methods. ‘Virtual anthropology’ might facilitate an increased understanding and appreciation of the range of human variation than possible with traditional skeletal collections and traditional methodologies, such as classic osteometric parameters.

This study does not specifically and directly test the classic osteometric landmarks, namely the anterior superior- and posterior superior-iliac spine, the superior rim on the pubic symphysis and the inferior margin of the ischium [42]. However, by studying the variability over the entire pelvis on a point-by-point basis, the variability at those classic landmarks was inherently tested. Our study proves that at anatomical areas related to the classic osteometric landmarks, the variability on a point-by-point basis is such that it would not result in an error in linear distance of more than 2 mm between landmarks. This is because at each point, the variability is 0.7 mm or less on a 2 standard deviation (SD) level.

Virtual models therefore enable us to appreciate more complex patterns of variation, as we are not limited to the conventionally used point-to-point distances between anatomical landmarks, more commonly referred to as inter-distance landmarks (ILDs). The use of non-standard methodological approaches enables us to explore the range and pattern of human variation from a different angle and ultimately may lead to the development of innovative osteometric techniques. The benefit of this approach is illustrated by recent work which showed that integrating non-standard measurements provides more information for complex population structures where there is a high rate of immigration, migration and relaxed border controls [43]. Additionally, virtual models derived from clinical CT data enable us to develop new forensic anthropological techniques that are not based on biased skeletal databases.

## Conclusion

Virtual bone models segmented from CT images with full exposure levels or minimal slice thickness produce geometries that are more precise. However, the effects of image segmentation and varying imaging conditions have no practical effect on the use of 3D virtual models, of the human pelvis, from a forensic anthropological point of view. Therefore, virtually modelled pelves from segmented patient-derived CT scans are a sufficiently precise source for forensic anthropological methods and for creating a modern virtual skeletal database.

## Electronic supplementary material

ESM 1 (AVI 19404 kb)

ESM 2 (AVI 19376 kb)

ESM 3 (AVI 19376 kb)

ESM 4 (AVI 19336 kb)

ESM 5 (AVI 19404 kb)

ESM 6 (AVI 19376 kb)

ESM 7 (AVI 19404 kb)

ESM 8 (AVI 19461 kb)

ESM 9 (AVI 16712 kb)

ESM 10 (AVI 16712 kb)

ESM 11 (AVI 19404 kb)

ESM 12 (AVI 16712 kb)

## References

[1] Steyn M, Iscan MY (1998). Sexual dimorphism in the crania and mandibles of south African whites. Forensic Sci Int.

[2] Steyn M, Iscan MY (1997). Sex determination from the femur and tibia in south African whites. Forensic Sci Int.

[3] Steyn M, Patriquin ML (2009). Osteometric sex determination from the pelvis—does population specificity matter?. Forensic Sci Int.

[4] Bidmos MA, Dayal MR (2004). Further evidence to show population specificity of discriminant function equations for sex determination using the talus of south African blacks. J Forensic Sci.

[5] Patriquin ML, Loth SR, Steyn M (2003). Sexually dimorphic pelvic morphology in south African whites and blacks. Homo.

[6] L’Abbe EN, Loots M, Meiring JH (2005). The Pretoria bone collection: a modern south African skeletal sample. Homo.

[7] Kern KF (2006). T. Wingate Todd: Pioneer of modern American physical anthropology. Kirtlandia.

[8] Hunt DR, Albanese J (2005). History and demographic composition of the Robert J. Terry anatomical collection. Am J Phys Anthropol.

[9] Dayal MR, Kegley AD, Strkalj G, Bidmos MA, Kuykendall KL (2009). The history and composition of the Raymond a. Dart collection of human skeletons at the University of the Witwatersrand, Johannesburg, South Africa. Am J Phys Anthropol.

[10] Marks MK (1995). William M. Bass and the development of forensic anthropology in Tennessee. J Forensic Sci.

[11] (2017) Forensic Anthropology Center: Age, sex and ancestry distibution of WM bass donated skeletal collection. The University of Tennessee, Knoxville: Forensic Anthropology Center

[12] Slice D, Untereggre C, Schaefer K, Bookstein F (2004). Modeling the precision of landmark data. Am J Phys Anthropol.

[13] Middleton A, Alminyah A, Apostol MA et al. Positional statement forensic odontology radiography and imaging in disaster victim identification positional statement of the members of the disaster victim identification working group of the International Society of Forensic Radiology and Imaging. J Forensic Radiol Imaging. doi: 10.1016/j.jofri.2016.08.003

[14] Maat GJR, Maes A, Aarents MJ, Nagelkerke NJD (2006). Histological age prediction from the femur in a contemporary Dutch sample—the decrease of nonremodeled bone in the anterior cortex. J Forensic Sci.

[15] Rissech C, Estabrook GF, Cunha E, Malgosa A (2006). Using the acetabulum to estimate age at death of adult males. J Forensic Sci.

[16] Hens SM, Rastelli E, Belcastro G (2008). Age estimation from the human os coxa: a test on a documented Italian collection*. J Forensic Sci.

[17] Eliopoulos C, Lagia A, Manolis S (2007). A modern, documented human skeletal collection from Greece. Homo.

[18] Gapert R, Black S, Last J (2009). Sex determination from the occipital condyle: discriminant function analysis in an eighteenth and nineteenth century British sample. Am J Phys Anthropol.

[19] Cardoso HF (2006). Brief communication: the collection of identified human skeletons housed at the Bocage museum (National Museum of Natural History), Lisbon, Portugal. Am J Phys Anthropol.

[20] Maat GJR. (2002) Citizens buried in the ‘Sint Janskerkhof’ of the ‘Sint Jans’ cathedral of ’s-Hertogenbosch in The Netherlands, ca. 1450 and 1830–1858 AD. In: Anthropologica Bs, ed. Leiden, pp. 1–62

[21] Lemmers, SAM, Schats R, Hoogland, MLP, Water-Rist AL. (2013) Fysisch antropologische analyse Middenbeemster. In: Hakvoort A, ed. De begravingen bij de Keyserkerk te Middenbeemster. pp. 35–60

[22] Baetsen S. (2001) Graven in de Grote Kerk, het fysisch-antropologisch onderzoek van de graven in de St. Laurens kerk van Alkmaar. Alkmaar Rapporten over de Alkmaarse Monumentenzorg en Archeologie

[23] van der Merwe AE, Morris AG, Steyn M, Maat GJR (2013) African skeletal remains housed at the Anatomisch Museum of the Leids Universitair Medisch Centrum. South African Archaeological Society Goodwin Series 11

[24] Danubio ME, Sanna E (2008). Secular changes in human biological variables in western countries: an updated review and synthesis. J Anthropol Sci.

[25] (2004) The Humna Tissue Act. In: Goverment UK, ed

[26] Stull KE, Tise ML, Ali Z, Fowler DR (2014). Accuracy and reliability of measurements obtained from computed tomography 3D volume rendered images. Forensic Sci Int.

[27] Decker SJ (2010) The human in 3D: advanced morphometric analysis of high-resolution anatomically accurate computed models. University of South Florida

[28] Verhoff MA, Ramsthaler F, Krahahn J (2008). Digital forensic osteology—possibilities in cooperation with the Virtopsy project. Forensic Sci Int.

[29] Franklin D, Cardini A, Flavel A (2013). Concordance of traditional osteometric and volume-rendered MSCT interlandmark cranial measurements. Int J Legal Med.

[30] Starck G, Lonn L, Cederblad A, Forssell-Aronsson E, Sjostrom L, Alpsten M (2002). A method to obtain the same levels of CT image noise for patients of various sizes, to minimize radiation dose. Br J Radiol.

[31] Crespo C, Rissech C, Thomas R, Juan A, Appleby J, Turbon D (2015). Sexual dimorphism of the pelvic girdle from 3D images of a living Spanish sample from Castilla-La Mancha. Homo.

[32] Franklin D, Cardini A, Flavel A, Marks M (2014) Morphometric analysis of pelvic sexual dimorphism in a contemporary Western Australian population. 128:72 10.1007/s00414-014-0999-8 24789357

[33] Decker SJ, Davy-Jow SL, Ford JM, Hilbelink DR (2011). Virtual determination of sex: metric and nonmetric traits of the adult pelvis from 3D computed tomography models. J Forensic Sci.

[34] Lopez-Alcaraz M, Garamendi Gonzalez PM, Aleman Aguilera I, Botella Lopez M (2013) Image analysis of pubic bone for sex determination in a computed tomography sample. 127:55. 10.1007/s00414-013-0900-1 23979057

[35] Clavero A, Salicru M, Turbon D (2015) Sex prediction from the femur and hip bone using a sample of CT images from a Spanish population. 129:83. 10.1007/s00414-014-1069-y 25270588

[36] Dobbe JGG, Strackee AW, Schreurs R (2011). Computer-assisted planning and navigation for corrective distal radius osteotomy, based on pre- and intraoperative imaging. IEEE Trans Biomed Eng.

[37] Ibánes L, Schroeder W. (2003) The insight segmentation and registration toolkit, software guide, Kitware Inc

[38] Carelsen B, Jonges R, Strackee SD (2009). Detection of in vivo dynamic 3-D motion patterns in the wrist joint. IEEE Trans Biomed Eng.

[39] Lorensen WE, Cline HE (1987). Marching cubes: a high resolution 3D surface construction algorithm. Computer Graphics and interactive techniques.

[40] Shahidi R. (1996) Surface rendering versus volume rendering in medical imaging: techniques and applications. 7th IEEE Visualization Conference

[41] Biwasaka H, Aoki Y, Tanijiri T et al. (2009) Analyses of sexual dimorphism of contemporary Japanese using reconstructed three-dimensional CT images—curvature of the best-fit circle of the greater sciatic notch. 11:S262. 10.1016/j.legalmed.2009.02.052 19362871

[42] Langley NR, Jantz LM, Ousley SD, Jantz RL, Milner G (2016). Data collection procedures for forensic skeletal material 2.0.

[43] Spradley KM, Jantz RL (2016). Ancestry estimation in forensic anthropology: geometric morphometric versus standard and nonstandard interlandmark distances. J Forensic Sci.

